# Prevention of Blindness in Stickler Syndrome

**DOI:** 10.3390/genes13071150

**Published:** 2022-06-26

**Authors:** Philip Alexander, Martin P. Snead

**Affiliations:** 1 NHS England Stickler Syndrome Highly Specialised Service, Cambridge University Hospitals NHS Foundation Trust, Cambridge CB2 0QQ, UK; mps34@cam.ac.uk; 2 Vitreoretinal Service, Addenbrooke’s Hospital, Hills Road, Cambridge University Hospitals NHS Foundation Trust, Cambridge CB2 0QQ, UK; 3 Vitreoretinal Research Group, John van Geest Centre for Brain Repair, University of Cambridge, Forvie Site, Cambridge CB2 0PY, UK

**Keywords:** retinal detachment prophylaxis, cryotherapy, laser retinopexy, giant retinal tear, stickler syndrome, COL2A1, COL11A1

## Abstract

Stickler syndromes are inherited conditions caused by abnormalities of structural proteins in the eye, inner ear and cartilage. The risk of retinal detachment, particularly due to the development of giant retinal tears, is high. Stickler syndrome is the most common cause of childhood retinal detachment. Although retinal detachment surgery in the general population has a high success rate, outcomes from surgical repair in Stickler syndrome patients are notoriously poor, providing a strong argument for prophylactic intervention. Variable case selection, absence of molecular genetic sub-typing and inconsistent treatment strategies have all contributed to the historic uncertainty regarding the safety and efficacy of prophylactic treatment. This paper reviews the major published clinical studies that have evaluated different methods and strategies for prophylaxis. Based on the current body of literature, there is extremely strong evidence from cohort comparison studies demonstrating the efficacy and safety of prophylactic retinopexy to reduce, but not eliminate, the risk of retinal detachment in Stickler syndrome patients. It is vital that this body of evidence is provided to Stickler syndrome patients, to enable them to make their own fully informed choice about whether to receive prophylaxis for themselves and particularly on behalf of their affected children, to reduce the risk of retinal detachment.

## 1. Introduction

Stickler syndromes are hereditary vitreoretinopathies caused by abnormalities in structural proteins that are essential for the normal development of the eye, inner ear, and cartilage. Although originally thought to be a single disorder, at least 10 different subtypes of Stickler syndrome have now been defined, with Type 1 Stickler syndrome accounting for 80% of patients. The most common threat to vision in patients with Stickler syndrome is the risk of rhegmatogenous retinal detachment, which frequently affects both eyes and can occur in childhood. This article explores the rationale and evidence for preventative strategies against retinal detachment in patients with Stickler syndrome.

## 2. Methods

The PubMed database was searched for cohort studies and reports of novel techniques, investigating retinal detachment prophylaxis for patients with Stickler syndrome. Relevant articles were retrieved, and the authors then manually reviewed the reference lists of primary studies and review articles to retrieve additional articles. The last search was performed in February 2022. The literature search strategy was based on the patient, intervention, comparison and outcome principle. The search included, but was not limited to, combinations of the following terms: ‘Stickler Syndrome’, ‘COL2A1′, ‘COL11A1′, ‘cryotherapy’, ‘cryopexy’, ‘laser retinopexy’, ‘retinal detachment’, ‘prophylaxis’, ‘prevention’, ‘giant retinal tear’.

## 3. Is Prevention of Retinal Detachment Required?

The adage that “prevention is better than cure”, attributed to the Dutch philosopher Desiderius Erasmus, is now a fundamental principle of modern healthcare policy [1]. In contrast, Karl Popper argued that “In the realm of errors, cure is better than prevention”, the corollary being that prevention of disease can only be applied in well-understood, homogenous conditions [2]. In his book, which tackles the dilemma of prevention versus cure, Christopher Dye argues that the most acceptable preventative strategies are those that are low-cost, high-efficacy methods for preventing a large, probable and imminent threat to health [3].

If retinal detachments could be consistently repaired with a high degree of anatomical and visual success, there would be a valid argument that prophylaxis is unnecessary.

Surgical repair of rhegmatogenous retinal detachment is highly effective in the general population. Anatomical success rates after one operation are 80–90% [4,5], with success rates well over 90% in some centres [6]. However, retinal detachment in Stickler syndrome is more complex and difficult to manage, and success rates for retinal detachment repair in patients with Stickler syndrome are much lower. Stickler syndrome is the commonest cause of retinal detachment in children, and paediatric retinal detachments characteristically present late, resulting in higher rates of macula-off detachment, proliferative vitreoretinopathy and poor visual acuity at presentation [7]. In the series described by Abeysiri et al. of Stickler syndrome patients with retinal detachment presenting to a large centre, more than 20% of the patients had inoperable retinal detachment at presentation, and of the patients that underwent surgery, one-third had bilateral retinal detachment, and primary success was achieved in just 50% (14/28) of patients [8].

Similar outcomes were achieved by Lee et al., who reported a 55% primary reattachment rate in patients with Stickler syndrome [9]. Read et al. studied children with Stickler syndrome presenting with retinal detachment and found that final anatomical success was achieved in 60% of them, with 20% of the patients resulting in either enucleation or phthisis [10]. Visual outcomes were correspondingly poor, with just 30% of the patients achieving 20/200 or better.10 In the series by Wubben et al., five of six eyes became phthisical despite surgical intervention [11].

Given that the lifetime risk of retinal detachment in Stickler syndrome is so high [12] and that the success of retinal detachment repair in these patients is poor, there is a strong argument for the use of prophylactic intervention.

## 4. Which Stickler Syndrome Patients Should Receive Prophylaxis?

Stickler syndrome, first described by Gunnar Stickler as a hereditary arthro-ophthalmolopathy, was originally thought to be a single-gene disorder. However, it is now known to encompass at least 10 different subtypes, with likely further genetic heterogeneity still to be resolved. Some of the early papers that discuss prophylaxis in Stickler syndrome refer to the Wagner–Stickler syndrome [13,14], because Wagner syndrome was at one time considered synonymous with the ocular-only variety of Stickler syndrome [15]. It is now known that Wagner syndrome is caused by mutations in the VCAN (5q13-q14) gene, has no systemic features, and is a completely separate disorder (OMIM #143200) from Stickler syndrome [16].

Early studies of prophylactic treatment for Stickler syndrome do not provide any details of genetic testing [13,14]. In all reports where genetic confirmation of Stickler syndrome was conducted prior to performing surgical prophylaxis [11,12,17,18,19], the patients had Type 1 Stickler syndrome, caused by mutations in COL2A1. Type 1 disease accounts for around 80% of all cases of Stickler syndrome and represents the majority of cases seen by ophthalmologists [16]. Type 2 Stickler syndrome patients also have a high risk of retinal detachment, but it is unclear whether the risk is as high as in the Type 1 Stickler syndrome patients [20], and prophylaxis in Type 2 patients has been much less studied [21].

## 5. How Should Prophylactic Treatment Be Performed in Stickler Syndrome?

A summary of the literature for prophylactic strategies in Stickler syndrome is shown in Table 1. All of the published studies are retrospective, and there is considerable variability in prophylaxis strategies and methods.

In 1994, Monin et al. described 22 patients with “Wagner–Stickler” syndrome who had developed a retinal detachment [14]. Of these, 10 patients received “peripheral confluent laser photocoagulation” in the fellow eye, but 5 developed retinal detachment. Four patients, receiving cryotherapy, vitrectomy, or “focal or circular” laser photocoagulation posterior to the equator, also developed retinal detachment. A further eight patients were treated with an encircling scleral buckle, but none of these patients developed detachment.

Alshahrani et al. described their experience of retinal detachment repair in 70 patients with Stickler syndrome and observed that 44 patients (62.8%) had had previous prophylactic laser therapy. The authors’ conclusion is that prophylaxis is not helpful in these patients, but none of these patients had had genetic testing to confirm the diagnosis, and only 22.6% of the patients had a family history, which is lower than would be expected. There was no control group, and there no details were provided about where or how the laser was applied. Due to these study limitations, it is impossible to make any assessment on the efficacy of prophylactic laser treatment from this study.

Leiba et al. described 10 patients from a family of 42 members with genetically confirmed Type 1 Stickler syndrome, who received one of two types of prophylactic laser treatment. Patients with extensive peripheral retinal degeneration with lattice degeneration in three or more retinal quadrants received 4–8 rows of encircling laser burns at the junction between the posterior border of the lesions and the unaffected retina, with extension of the laser barrier (2–3 rows) to include isolated areas of lattice degeneration at or posterior to the equator. In eyes with only small, localised lesions of lattice degeneration or isolated breaks, only focal treatment was applied, with visibly abnormal areas encircled with 3–6 rows of laser burns. This group found that there was a significantly higher incidence of retinal detachment in non-lasered eyes compared to lasered eyes.

**Figure 1:**
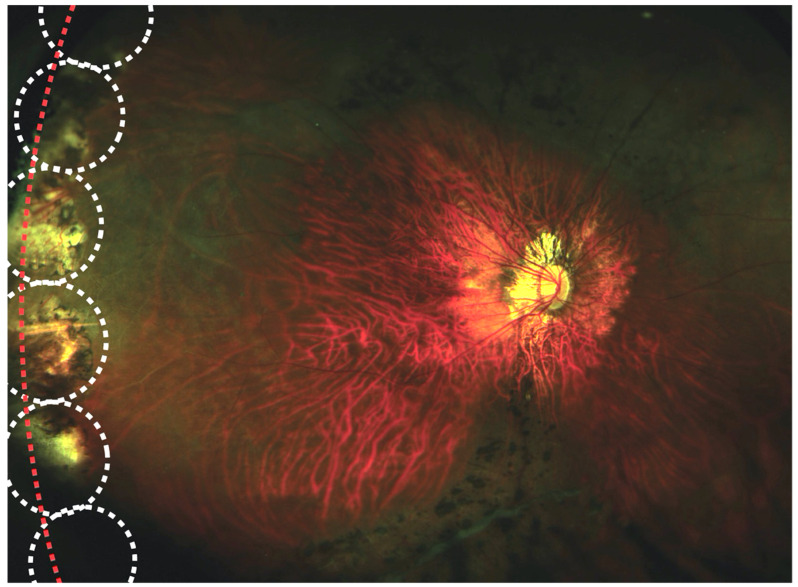
Prophylactic 360-degree cryoretinopexy in Type 1 Stickler syndrome according to Cambridge Prophylactic Cryotherapy Protocol. White circles show locations of individual cryotherapy applications, which are contiguous with one another and include the ora serrata (red line).

Ang et al., based in Cambridge UK, were the first to describe a standard protocol for applying retinopexy in “high-risk” genetically confirmed type 1 Stickler syndrome patients, using monitored transconjunctival cryotherapy applied in a contiguous fashion to the post-oral retina, with the specific objective of preventing elevation and progression of the posterior flap of a giant retinal tear, should it occur at the time of posterior vitreous detachment (Figure 1). This treatment, now known as the Cambridge Prophylactic Cryotherapy Protocol”, was offered to all Type 1 Stickler syndrome patients with eyes unaffected by retinal detachment, irrespective of the presence or absence of lattice degeneration. Eyes treated prophylactically exhibited a much lower prevalence of retinal detachment, and importantly, no patients receiving bilateral prophylaxis developed bilateral retinal detachment. The authors acknowledged the difference in mean ages and follow-up durations between the study and the control groups. In a subsequent paper by the Cambridge group [12], the limitations of their first study were addressed by matching study and control patients not only by age but also by follow-up duration. This study was intentionally biasing against the benefit of treatment, to ensure that any true treatment effect of the Cambridge Prophylactic Cryotherapy Protocol would be underestimated. The study, which included 487 patients with Type 1 Stickler syndrome, found that patients with no prophylaxis in either eye had a 5.0-fold increased risk of retinal detachment compared to the matched bilateral prophylaxis group. For patients who had already had retinal detachment in one eye, the risk of retinal detachment in the fellow eye was 8.4-fold compared to fellow eyes receiving prophylaxis. This study remains the largest case series of prophylactic treatment for Stickler syndrome in the literature (and indeed is larger than the rest of the world literature combined), has the longest follow-up and presents powerful evidence in favour of prophylactic cryotherapy in patients with Stickler syndrome.

Wubben et al. described 15 patients with genetically confirmed Type 1 Stickler syndrome. The mean follow-up time was 6.4 years (range, 4 months–16 years). The authors did not describe their laser prophylaxis technique but found that the risk of developing retinal detachment was only 5% in eyes receiving laser prophylaxis (1/20) compared to 50% in eyes not receiving laser prophylaxis (5/10). Of interest was the very poor outcome in patients developing retinal detachment—five of the six eyes became phthisical despite surgical intervention.

Ripandelli et al. reported a single-surgeon series of 52 patients with genetically confirmed Type 1 Stickler syndrome, who had developed retinal detachment in one eye and therefore received prophylaxis with a 6 mm scleral encircling band in the fellow eye. The rationale for this technique was to reduce vitreoretinal traction, and mean follow-up was 15.6 years, with a minimum of 12 years in all cases. In 39/52 eyes, cryotherapy retinopexy was also performed due to the presence of retinal tears, retinal holes and/or lattice degeneration. The authors found that none of the patients receiving adjuvant cryotherapy developed retinal detachment, yet 5/13 eyes receiving scleral buckling, without associated cryotherapy, developed retinal detachment. This not only supports the argument for retinopexy but also indicates that relief of vitreoretinal traction is less important than preventing the development and/or progression of retinal breaks.

Morris et al. (2021) described a two-step prophylactic retinopexy in five eyes of four patients with type 2 Stickler syndrome [21]. Step 1 of the prophylaxis emulated the successful Cambridge strategy by applying moderately high-intensity burns in a tight grid pattern from the juxtaoral serrata and extending to 4 mm posteriorly, halfway to the vortex vein ampullae, to produce a “second ora”. Step 2 of the prophylaxis extended the laser grid posteriorly to beyond the line of the vortex vein ampullae to try to prevent the development of posterior tears [22]. In their series, none of the five treated eyes developed retinal detachment or retinal tear over the mean follow-up period of 8.7 years. There was an asymptomatic visual field constriction to an average of 50 degrees in each meridian. One eye developed pupillary mydriasis which persisted for 6 months before resolution. There was no epimacular proliferation in any of the treated eyes [22].

## 6. Laser vs. Cryotherapy Retinopexy

**Figure 2:**
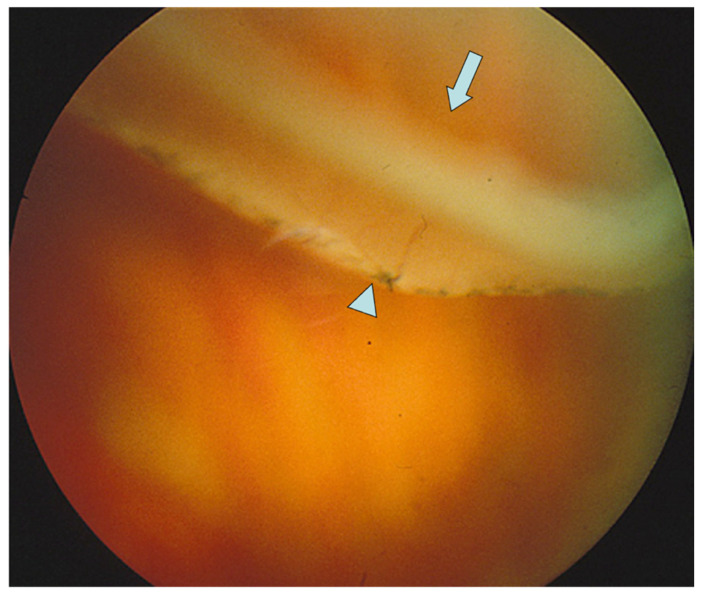
Retinal detachment due to a giant retinal tear in a patient type 1 Stickler syndrome. Note previous laser prophylaxis is too posterior to prevent detachment. Arrow = Giant retinal tear, arrow head = equatorial laser prophylaxis. Reproduced with permission from Snead, MP (2022): Retinal detachment in childhood. Chapter in: Paediatric Ophthalmology and Strabismus 6th Edition. Editors Lyons C & Hoyt C. Elsevier Saunders. In press.

**Figure 3:**
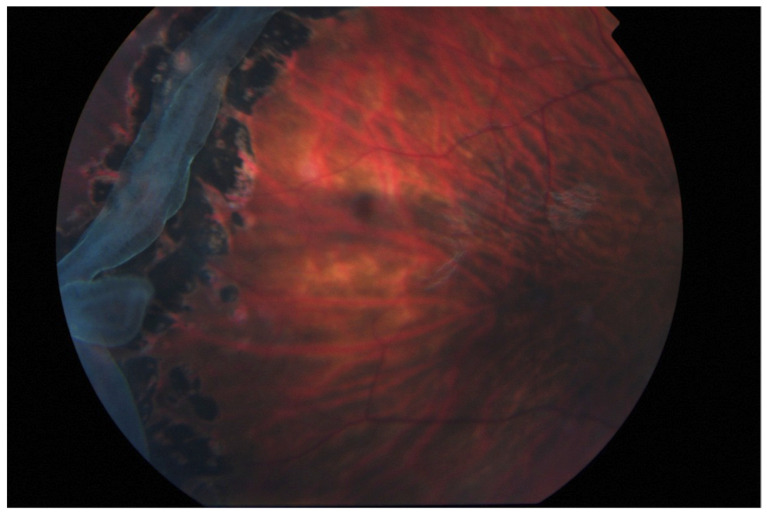
Laser retinopexy to arrest the progression of a giant retinal tear in type 1 Stickler syndrome (no previous prophylaxis). Reproduced with permission from Snead, MP (2022): Retinal detachment in childhood. Chapter in: Paediatric Ophthalmology and Strabismus 6th Edition. Editors Lyons C & Hoyt C. Elsevier Saunders. In press.

There has been no head-to-head comparative study to evaluate the efficacy of laser retinopexy versus cryotherapy retinopexy to prevent retinal detachment. The location of retinopexy within the retina is likely to be much more important than the modality of treatment (see Figure 2 and Figure 3). Some groups express a preference for barrage laser over cryotherapy because of the perceived increased inflammatory reaction associated with the latter. Cryotherapy of retinal breaks can cause dispersion of retinal pigment epithelial (RPE) cells within the vitreous [23] but avoids the tissue vaporisation that can occur with laser and can be used in the presence of compromised ocular media [24]. Both laser and cryotherapy cause a significant breakdown of the blood–retinal barrier [25]. A clinical trial comparing the two modalities for retinal detachment repair showed no difference in visual outcome [26].

Our group’s experience with cryotherapy prophylaxis has been overwhelmingly positive, with high efficacy, retention of good visual acuity, and no associated epiretinal membrane (ERM) formation. Cryotherapy is performed contiguously at the juxtaoral retina even in the absence of retinal breaks. Despite the experimental evidence that cryotherapy may enhance RPE cell dispersion into the vitreous cavity using giant or other retinal tears as a conduit, it is interesting to note that of the 964 eyes that have received cryotherapy prophylaxis under our care, none of the patients with successful prophylaxis developed a visually significant epiretinal membrane. One patient, whose prophylaxis failed, developed an epiretinal membrane requiring treatment (unpublished data). We also observed significant epiretinal membrane formation after retinal detachment in patients that did not receive prophylactic therapy. The association between retinal tears prior to treatment and ERM is well established.

Shapiro et al. astutely noted that prophylactic strategies are learned during training and therefore the choice of cryotherapy vs. laser is highly influenced by each surgeon’s particular educational lineage [24]. There may be a reluctance to perform 360-degree cryotherapy as per the Cambridge prophylactic cryotherapy protocol, because of a lack of exposure to training in performing this procedure [21,24].

## 7. Conclusions

This paper has reviewed the rationale and evidence for the prevention of retinal detachment in patients with Stickler syndrome. All of the studies on this topic were retrospective. However, the studies shown in Table 1 demonstrate an overwhelming support for the use of prophylactic retinopexy in these patients [11,12,17,18,19,21]. The two case series that reported no benefit after prophylaxis are less persuasive because of the poorly defined patient selection, unspecified treatment protocol and lack of a control group [14,22]. Despite this, some still question the effectiveness of prophylaxis in the absence of a randomised controlled trial. While randomised controlled trials would provide the best level of evidence, there is already proof of safety and efficacy from non-randomised, cohort comparison studies, and it is essential that Stickler syndrome patients (i) receive accurate genotyping to stratify their risk of RD and (ii) are provided with all of the information available, to enable them to make their own fully informed choice about whether to receive prophylaxis for themselves and particularly on behalf of their affected children. Late presentations of bilateral inoperable retinal detachment, especially in the context of hearing impairment and speech and mobility issues, have a devastating and life-long impact on the future development of these children.

## Figures and Tables

**Table 1:** Summary of studies evaluating strategies to prevent retinal detachment (RD) in patients with Stickler syndrome.

Author	Stickler Type (n)	Laser/Cryotherapy/Buckle	Type of Study	Follow Up	Results
Monin et al., 1994, Paris, France [14]	22 patients with Wagner–Stickler syndrome	Laser photocoagulation, or encircling scleral buckle in fellow eyes of patients with RD in the first eye	Retrospective case series (no control group)	Up to 5.5 years	50% of patients receiving laser treatment developed RD. None of the scleral buckle patients developed RD.
Leiba et al., 1996, Rehovat, Israel [17]	10 patients from a single family with genetically confirmed Type 1 Stickler syndrome. Untreated family members were used as controls during the study follow-up	Primary prophylactic laser photocoagulation, either (a) circumferentially, at the posterior border of retinal lesions, or (b) around areas of abnormal retina	Retrospective study	1–15 years	10% of lasered eyes developed retinal detachment, compared to 44% of non-lasered eyes
Ang et al., 2008, Cambridge, UK [18]	93 patients (155 eyes) with genetically confirmed Type 1 Stickler syndrome and 111 control patients (222 eyes) who did not receive any intervention	360-degree cryotherapy of the juxtaoral retina, for prevention of giant retinal tear	Retrospective comparative case series	Up to 33 years	With no retinopexy, 73% of the patients suffered RD, and 48% were bilateral. Of those receiving retinopexy, 8% developed RD, but none were bilateral.
Fincham et al., 2014, Cambridge, UK [12]	293 patients with genetically confirmed Type 1 Stickler syndrome and 194 control patients who did not receive any intervention	Cambridge Prophylactic Cryotherapy Protocol: 360-degree cryotherapy of the juxtaoral retina, for prevention of giant retinal tear	Retrospective comparative case series, matched for age and follow-up duration	1–36 years	The bilateral and unilateral control group had a 5.0-fold and 8.4-fold, respectively, increased risk compared to eyes receiving prophylaxis
Al-Shahrani et al., 2015, Riyadh, Saudi Arabia, [22]	70 eyes of patients with Stickler syndrome. Genetic testing not specified. No control group.	Details of prophylactic laser retinopexy not specified	Retrospective case series	1 week to 10 years	No genetic confirmation, no control group and no details of type of laser prophylaxis, so impossible to assess prophylaxis efficacy from this study.
Wubben et al., 2018, Ann Arbor, Michigan, USA [11]	15 patients with genetically confirmed Type 1 Stickler syndrome; of these, 20 eyes had prophylactic laser retinopexy	Laser (details not reported)	Retrospective comparative case series	4 months -16 years	5% risk of RD with prophylaxis; 50% risk of RD without prophylaxis
Morris et al., 2021, Birmingham, Alabama, USA [21]	5 eyes of 4 patients from a single family with confirmed Type 2 Stickler syndrome	Encircling grid laser (Modified Ora Secunda Cerclage)	Retrospective case series	3–12 years	0/5 eyes developed retinal tear or retinal detachment.
Ripandelli et al. (2022), Rome, Italy [19]	Fellow eyes of patients with genetically confirmed Type 1 Stickler syndrome who had had unilateral retinal detachment.	All eyes received a 6 mm-wide encircling band. Cryoretinopexy was performed on any retinal tears, holes or lattice degeneration	Retrospective case series	Mean 15.6 years, all >12 years	Scleral buckle without cryo: 5/13 developed RDScleral buckle with cryo: 0/39 developed RD
